# OA21 Are novel anti-IL5 biologics triggering rheumatoid arthritis?

**DOI:** 10.1093/rap/rkad070.021

**Published:** 2023-09-27

**Authors:** Nathan Dean, Ian Clifton, Rashad Salman, Dennis McGonagle

**Affiliations:** Leeds Teaching Hospital Trust, Leeds, United Kingdom; Leeds Teaching Hospital Trust, Leeds, United Kingdom; Leeds Teaching Hospital Trust, Leeds, United Kingdom; Leeds Teaching Hospital Trust, Leeds, United Kingdom; University of Leeds, Leeds, United Kingdom

## Abstract

**Introduction:**

Biologics are being increasingly used for the treatment of inflammatory diseases across a wide range of specialties. Of particular interest, it is well established that monoclonal therapy for one inflammatory disease may paradoxically trigger another inflammatory disease, for example TNF therapy triggering multiple sclerosis, and IL-17 therapy for psoriasis linked to inflammatory bowel disease. Here we report the case of a patient who developed anti-CCP positive rheumatoid arthritis soon after starting a novel anti-IL-5 biologic used to treat his severe eosinophilic asthma.

**Case description:**

This 77-year-old gentleman with a background of severe eosinophilic asthma presented to our early arthritis clinic 18 months after having been started on either mepolizumab or a long-acting IL-5 blocker as part of a clinical trial. Prior to starting biologics, his asthma had been poorly controlled with salbutamol, Fostair, tiotropium inhalers and low-dose corticosteroids.

Within weeks, he developed symmetrical arthralgias involving small joints, particularly his wrists and knuckles, as well as significant early morning stiffness lasting more than one hour. On examination, he had clinical synovitis in the wrists and metacarpophalangeal joints bilaterally, as well as right shoulder capsulitis with limiting range of motion.

Blood tests revealed a raised CRP of 24mg/L, WCC 8.77 10*9/L, rheumatoid factor of 263.2 iu/mL and anti-CCP antibody of > 300 U/mL. Ultrasound imaging of the hands and wrists bilaterally showed grade II grey scale with grade II power Doppler, and some erosions in the wrists bilaterally. There was hypoechogenicity of the left extensor carpi ulnaris tendon with some associated grey scale and power Doppler.

He was diagnosed with rheumatoid arthritis as per the American College of Rheumatology (ACR)/European League Against Rheumatism (EULAR) diagnostic criteria and started on prednisolone 10mg daily as a rescue therapy to reduce the inflammation, followed by sulfasalazine one month later as the disease modifying agent. He was followed-up in rheumatology clinic two months later and showed significant improvements: the pains and swellings of his joints had settled, and whilst he still gets early morning stiffness this is not so debilitating. His inflammatory markers had also resolved with CRP <5.0mg/L and WCC 9.31 10*9/L.

**Discussion:**

We report a case of new onset rheumatoid arthritis associated with the commencement of an anti-IL-5 biologics for severe asthma. To our knowledge, this has only been reported in the literature on a handful of occasions and in each case the patients were on oral corticosteroids with the majority being weaned off in parallel to developing the new symptoms [1,2]. As such there has been a challenge in associating the development of arthritis with the initiation of the anti-IL-5 biologic, as opposed to the withdrawal of steroids unmasking a pre-existing disease.

Emerging evidence has implicated a core role for regulatory eosinophils (rEos) in the resolution of rheumatoid arthritis (RA) [3]. In murine models of RA, an expansion of rEos in the synovium was sufficient to bring about remission of arthritis. Inhibiting the IL-5 pathway would subsequently induce relapse of the arthritis [3]. Further studies are required to characterise the significance of these findings in clinical cohorts and to identify whether there is an actual association between novel anti-IL-5 biologics and rheumatoid arthritis.

**Key learning points:**

Our improved understanding of the mechanisms underlying inflammatory diseases has led to the increased use of biological therapies in treating these conditions. However, these therapies may be paradoxically triggering other inflammatory diseases, particularly autoimmune rheumatological diseases.

Here we have identified a patient who developed anti-CCP positive rheumatoid arthritis soon after commencing a novel anti-IL5 for severe asthma. Mechanistically, this may be related to the depletion of regulatory eosinophils tipping the balance towards pro-inflammatory pathways in the synovial joints.

More work is required to identify whether this phenomenon is being more widely observed in clinical practice, and if so how we can best manage it going forwards.

 
